# A promising future for tele-mental health in Oman: A qualitative exploration of clients and therapists’ experiences

**DOI:** 10.1192/j.eurpsy.2025.1418

**Published:** 2025-08-26

**Authors:** T. Al-Mahrouqi, K. Al-Alawi, M. Al-Alawi, N. Al Balushi, A. Al Ghailani, H. Al Sabti, H. Al Sinawi

**Affiliations:** 1 Oman Medical Speciality Board; 2 Sultan Qaboos University Hospital, Muscat, Oman

## Abstract

**Introduction:**

**Objectives:** Tele-mental health services can play an important role in overcoming barriers in mental health services in the Eastern Mediterranean Region. However, despite its potential, tele-mental health has not been widely adopted in Oman.

**Objectives:**

This study is an exploratory investigation into the experiences of therapists and their clients in utilizing video-based tele-mental health care during the COVID-19 pandemic.

**Methods:**

A total of 19 semistructured qualitative interviews were individually conducted, it included 13 adult clients with mental health conditions who received video-based tele-mental health care and six clinical psychologists who provided video-based tele-mental health care during the COVID-19 pandemic.

**Results:**

The clients reported favorable experiences using tele-mental health, with the primary benefits being convenience, easy accessibility to subspecialized care, reduced absenteeism from work with commuting costs, and alleviated mental health stigma. The therapists also expressed experiencing benefits from tele-mental health, such as reduced risk of intrahospital infection, reduced healthcare costs, and the achievement of work-life balance. Primary concerns were related to the lack of public tele-mental health services, lack of specified tele-mental health guidelines, shortage of trained therapists, limited access to high-speed Internet, electronic devices, privacy, and concerns toward the security of telehealth systems in general.

**Conclusions:**

Clients and therapists report that tele-mental health offers new opportunities to improve the quality of mental healthcare services in Oman, and that the challenges could be resolved by establishing governmental tele-mental health services along with developing tele-mental health guidelines and implementing local postgraduate clinical psychology programs in universities in Oman.

**Disclosure of Interest:**

None Declared

